# Evaluation of the efficacy of ProHeart^®^ 6 (moxidectin) against a resistant isolate of *Dirofilaria immitis* (JYD-34) in dogs

**DOI:** 10.1186/s13071-017-2431-y

**Published:** 2017-11-09

**Authors:** Dwight D. Bowman, Tom L. McTier, Eric L. Adams, Sean P. Mahabir, Joyce A. Login, Tara Bidgood, Debra J. Woods

**Affiliations:** 1 Department of Microbiology and Immunology, College of Veterinary Medicine, Cornell University, 930 Campus Road, Ithaca, NY 14853 USA; 2 Zoetis, Kalamazoo, MI USA; 3 Northern Biomedical Research, Inc., Norton Shores, MI USA; 4 Zoetis, Parsippany, NJ USA

**Keywords:** ProHeart^®^ 6, Moxidectin, Macrocyclic lactone, Canine heartworm, *Dirofilaria immitis*, Resistance

## Abstract

**Background:**

In a previous study, it was demonstrated that ProHeart^®^ 6 (PH6) (moxidectin, Zoetis) provided only about 20% efficacy in a small six-dog study against a macrocyclic lactone –resistant *Dirofilaria immitis* isolate (Jd2009–2) when dogs were inoculated with infective third-stage larvae (L3) at the end of the dosing period (ie, 180 days post treatment). The objective of the current study was to determine the prophylactic efficacy of a moxidectin sustained-release formulation (PH6) against a confirmed macrocyclic lactone–resistant isolate of *D. immitis* (JYD-34) in dogs when administered by subcutaneous injection at the labeled dose of 0.17 mg/kg 2 days before L3 inoculation. This was intended to model the scenario where dogs become infected with resistant heartworms at the end of the PH6 treatment period (ie, 6 months post treatment) when dogs would routinely be given another injection under normal field use.

**Methods:**

Twelve purpose-bred Beagle dogs (six males and six females) were selected and randomly allocated to two groups, untreated controls and PH6-treated dogs in groups of six each. The dogs were ≥8 months old at the start of the study, and using blood samples collected on Day −7 were shown to be negative for adult heartworm antigen and microfilariae. On Day 0, the dogs in the untreated control group were administered saline subcutaneously by injection, and the dogs in the treated group were administered PH6 according to label instructions. On Day 2, each dog was inoculated in the inguinal area with 50 L3 of *D. immitis*. The dogs were necropsied on Day 150 (148 days post infection), and the worms were collected and counted.

**Results:**

All of the six control dogs were infected and harbored a range of 21 to 37 worms (geometric mean, 25.4; 10.9 males and 13.9 females). Only one of the six PH6 dogs was found to be infected, harboring a single male worm. Efficacy was 99.5% (geometric mean).

**Conclusion:**

ProHeart^®^ 6 was highly effective in preventing the development of heartworms in dogs challenged with a confirmed macrocyclic lactone–resistant heartworm isolate (JYD-34) 2 days prior to treatment.

## Background

ProHeart^®^ 6 (moxidectin, Zoetis) (PH6) is the only heartworm preventive in the United States that is designed to provide protection against heartworm infection, ie, infection with *Dirofilaria immitis*, for an extended period. All the other products that are available are dosed such that they provide protection by killing third-stage (L3) and the developed fourth-stage larvae (L4) that have been acquired by a dog over the previous 30 days. Thus, with the products that are administered monthly, the treatment kills the larvae from the mosquitoes that have entered the dogs and which have developed anywhere from 1 to 30 days before treatment. PH6, through its sustained-release formulation, provides prevention by maintaining levels of the active preventive molecule, moxidectin, at levels that kill incoming larvae for 180 days that, at the time of approval, supplied 100% efficacy [1].

Work that was first reported in 2013 and published in 2015 tested PH6 against a presumed resistant field isolate, Jd2009, of canine heartworm that had been transferred as L3 s from mosquitoes from an original source dog in Earle, Arkansas, USA [2, 3]. In this study, six dogs were infected with L3 of the Jd2009 isolate 180 days after drug administration. This work only utilized four treated dogs and two control dogs, but showed that unlike when the product was originally approved with 100% of the dogs being protected from heartworm infection 6 months post treatment, all four treated dogs had adult heartworms when examined 5 months after infection. The numbers were reduced compared with the control dogs, but with the small group size, statistics were not applied to the data. Thus, Jd2009 appeared to be a resistant isolate that was capable of developing into adults in dogs if they became infected at the end of the 6-month protective period supplied by the product.

The study with Jd2009 was performed to verify that there were resistant isolates present that would develop in dogs when they were tested in the same manner as when they were originally approved. In the case of PH6, this meant that the dogs were inoculated with L3 6 months after a single injection. If a dog is receiving PH6 twice a year, it is assumed the dog would be redosed with PH6 near the 6-month target date, but not exactly 180 days after the last treatment. The objective of the current study was to examine whether a known resistant isolate would successfully develop in dogs if they were to receive the heartworm infection closer to the actual administration of the initial PH6 treatment or closer to the date of the expected follow-up treatment.

## Methods

### Ethical approval

The study was approved by the facility Institutional Animal Care and Use Committee (IACUC) with the concurrence of Zoetis and followed the guidelines of VICH GL19 [4]. Masking of the study was assured through the separation of functions. All personnel conducting observations or animal care or performing infestations and counts were masked to treatment allocation. The protocol for this study was approved by the Zoetis IACUC, and the study was conducted in accordance with state and national/international regulations regarding animal welfare.

### Animals and design

The study included 12 purpose-bred beagles (six males and six females) from Ridglan Farms (Mt. Horeb, Michigan, USA). Each animal was positively and uniquely identified by an ear tattoo. At the beginning of the study, the animals were between 15 and 22 months of age. Each animal was housed individually in pens constructed of metal mesh and/or stainless-steel frames. Feed was offered to the dogs once per day, and water was available ad libitum.

General health was observed by appropriately trained personnel once daily throughout the study; the day the dogs were scheduled for treatment with PH6 was assigned as being Day 0. All 12 dogs received physical examinations by an experienced veterinarian 5 days before they received the treatment with PH6. On Day 0, dogs were assessed for overall health, and again at 3 and 6 h after treatment administration, and then again on Day 1, 24 h after PH6 treatment administration.

All dogs were weighed on Day −4 and then weekly, and the body weight (kg) was recorded with the weight being monitored to verify that they were maintaining body weights such that they remained within the original treatment weight bands until the end of the trial.

Blood samples from each animal were collected in potassium ethylenediaminetetraacetic acid (EDTA) tubes on Day −6 and Day 120. These samples were examined for the adult *D. immitis* antigen and for microfilariae (MF). Day 120 examination was conducted to detect heartworm infections acquired prior to selection for the study that were not detectible on the blood examination performed on Day −6.

The study followed a randomized complete block design and block was based on Day −4 body weights, isolate, treatment, pen, and room. At randomization the dogs were assigned to either the placebo group (T01) or to the PH6-treated group (T02).

### Treatment

The dogs were treated with PH6 as per label instructions as appropriate for their body weight.

### Isolate and infection with *D. immitis*

The isolate used in this study was JYD-34. For the original JYD-34 isolate, a blood sample was collected from a heartworm microfilariae-positive dog originally from Pittsfield, Illinois, USA that was discovered to have a patent heartworm infection of unknown duration. The dog had previously received at least one dose of Ivomec^®^ (ivermectin, Merial; actual dose not known) before the original isolate sample was collected, but no additional documented macrocyclic lactones (MLs) were given again to this dog. The blood sample was sent to TRS Labs, Athens, Georgia, USA on July 12, 2010, and it was used on July 13, 2010 to infect mosquitoes. Recipient dogs at TRS Labs were infected with larvae from mosquitoes on July 29, 2010. The age of the original heartworm infection is unknown. The JYD-34 isolate was validated in April 2011 with dogs inoculated with L3 s on July 29, 2010 testing positive for MF and adult heartworm antigen (DiroCHEK^®^, Zoetis).

On Day 2, 12 dogs were administered 50 viable *D. immitis* L3 (JYD-34 isolate) by subcutaneous injection in the inguinal region. Zoetis personnel provided the infected mosquitoes, harvested the larvae, and performed the inoculations.

### Necropsies and worm counts

All dogs were euthanized humanely on Day 150. At the time of euthanasia, each dog was given 2 mL of heparin (1000 USP units/mL) intravenously prior to a lethal dose of pentobarbital euthanasia solution. After euthanasia, the pleural and peritoneal cavities were examined for adult *D. immitis* worms, and the posterior and anterior venae cavae were clamped before removal of the heart and lungs. The precava, right atrium, right ventricle, and pulmonary arteries (including those coursing through the lungs) were dissected and examined.

### Data analysis

The experimental unit for treatment was the individual dog. Prior to statistical analysis, worm counts were natural log transformed {log_e_(x + 1)}. The statistical model for log-transformed live heartworm counts was a mixed linear model. The model contained the fixed effects of treatment and the random effects of room, block within room, and error. Least squares means and standard errors were calculated and 95% confidence intervals were constructed for each treatment. Geometric means (back-transformed means) were calculated from the least squares means and corresponding back-transformed 95% confidence intervals were reported, along with minimum and maximum values for the raw data. Treatment differences between each group were assessed at the two-tailed 5% level of significance (*p* < 0.05). Percent reduction in worm count for the treatment groups were estimated using the following formula:

$$ \mathrm{Geometric}\  \mathrm{Mean}\%\mathrm{Efficacy}=100\ \mathrm{x}\ \frac{\left[\ \mathrm{Geometric}\  \mathrm{mean}\  \mathrm{count}\ \left(\mathrm{placebo}\right)\hbox{--} \mathrm{Geometric}\  \mathrm{mean}\  \mathrm{count}\ \left(\mathrm{treated}\right)\ \right]}{\left[\ \mathrm{Geometric}\  \mathrm{mean}\  \mathrm{count}\ \left(\mathrm{placebo}\right)\ \right]} $$

The prevention rates for treatments were estimated by percentage of animals without heartworms from the necropsy on Day 150.

## Results

All tests were negative for both MF and heartworm antigen on both test days. No adverse events were observed for any animal on study.

All six of the placebo-treated dogs (T01) were found to be infected at necropsy; a single treated dog had a single male worm at necropsy (Table 1). Adult *D. immitis* counts for the placebo group (T01) ranged from 21 to 37, with a geometric (arithmetic) mean of 25.4 (25.8). In the PH6 group (T02), adult *D. immitis* counts ranged from 0 to 1, and geometric (arithmetic) mean counts were 0.1 (0.2). Percentage reduction in the geometric (arithmetic) mean count compared with placebo (T01) was 99.5% (99.4%).

**Table 1:** Live adult heartworm counts: Individual dog counts with means, percent reductions, and statistical comparisons. Dogs each inoculated with 50 L3 (JYD-34 isolate) on Day 2 and necropsied on Day 150 (148 days PI)

Group	Animal ID	Live Adult Worm Counts		
		Male	Female	Total
T01 Saline Control 0 mg/kg	HUC-4	8	14	22
	QQY-3	14	9	23
	TEB-4	10	17	27
	PED-4	12	25	37
	RCB-4	14	11	25
	XRQ-3	9	12	21
	Arithmetic Mean:	11.2	14.7	25.8
	Geometric Mean:	10.9	13.9	25.4
T02 ProHeart^®^ 6 Single Treatment Day 0	FHA-4	1	0	1
	ZEB-4	0	0	0
	YZX-3	0	0	0
	YKB-4	0	0	0
	RVW-3	0	0	0
	PIW-3	0	0	0
	Arithmetic Mean:	0.2	0.0	0.2
	Geometric Mean:	0.1	0.0	0.1^a^
% Reduction Arithmetic Mean:		98.5	100.0	99.4
% Reduction Geometric Mean:		98.9	100.0	99.5

^a^Statistical comparison were made on geometric means only; PH*®* 6 mean statistically different form the control mean (*P* < 0.0001)

## Discussion

In this study, PH6 was 99.5% efficacious against the JYD-34 isolate when treatment was initiated at the same time as L3 inoculation. In previous studies the JYD-34 isolate was found to have been refractory to three different prevention products approved for use in the United States [Heartgard^®^ Plus (ivermectin/pyrantel, Merial), Trifexis^®^ (spinosad + milbemycin oxime, Elanco), Revolution^®^ (selamectin, Zoetis)] [5]. In these studies, even three monthly treatments of Revolution^®^, Heartgard^®®^ Plus, and Trifexis^®^ were not protective. In the approval of NexGard Spectra^®^ (afoxolaner/milbemycin oxime, Merial) in the EU, this product, which contains milbemycin oxime at the same dose as in the products in the United States, had an efficacy of only 70% against JYD-34, even after six repeated monthly treatments [6]. Follow-up studies have confirmed phenotypic and genetic resistance of heartworms to MLs in the United States [3, 7]. Nevertheless, MLs are the only class of drugs available for use as heartworm preventives, and thus will require judicious use going forward to provide the best heartworm prevention possible. Additional work needs to be done to profile ML preventive products against various resistant heartworm isolates.

There was discussion before the current study’s initiation as to whether the treatment should occur before the inoculation, at the same time as the inoculation, or after the inoculation. Based on product pharmacology, L3 s given to dogs a couple days before treatment would see slightly higher levels of product and those given to a dog a couple days after treatment would see slightly lower levels of product. To simulate the efficacy of PH6 at re-treatment, the optimum study design would have been to treat the dogs, wait until just before re-treatment (~180 days), inoculate with L3 s, and then re-treat with PH6. This would have lengthened the study, however, by an additional 6 months and it was felt that this was unwarranted because at 180 days after treatment the product is fully effective against incoming susceptible L3 s as demonstrated by the label claims [1]. Thus, the dogs were treated, and then 2 days later, they were inoculated with heartworm L3 s. This accomplished two purposes: 1) reduced the length of the study by 6 months and 2) allowed some drug to be within the animals when the larvae were inoculated. In this scenario, the larvae were exposed to slightly less drug than would have been present if the PH6 was administered at the same time or after the L3 s were administered. It was thought that this also mimicked well what would happen at the end of 6 months when a dog would return to the clinic with sufficient moxidectin still being present to be fully effective in preventing susceptible L3 s from maturing.

In the face of emerging heartworm resistance, compliance should be viewed as being ever more important. Proper dosing of heartworm-negative animals with preventive medications will likely help reduce the spread of heartworm resistance. Lack of effectiveness (LOE) of heartworm preventive products may result from failure to administer the products correctly. This is especially important for the monthly products that are administered by the pet owners at home where compliance is often problematic. ProHeart^®^ 6, while removing the concerns with LOEs due to owner compliance issues and having demonstrated efficacy against a known resistant isolate of *D. immitis*, should be considered as an important tool for the veterinarian in the ongoing fight against heartworm disease.

## Conclusions

ProHeart^®^ 6 was highly efficacious (99.5%) against the JYD-34 isolate of *Dirofilaria immitis* when administered near the time of L3 inoculation.
